# Clinical and Functional Outcomes of Delta Large-Channel Endoscopic Lumbar Decompression: A Systematic Review and Meta-Analysis

**DOI:** 10.3390/brainsci16070731

**Published:** 2026-07-11

**Authors:** Rishi Jain, Nikhil Sriram, Mehul Mittal, David Zhang, Noah B. Drewes, Dillan Prasad, James M. Mossner, Nader S. Dahdaleh, Najib El Tecle, Christopher S. Ahuja

**Affiliations:** 1 Department of Neurological Surgery, Northwestern University Feinberg School of Medicine, Chicago, IL 60611, USA; rishi.jain@northwestern.edu (R.J.);; 2 Department of Surgery, Division of Neurosurgery, Southern Illinois University School of Medicine, Springfield, IL 62702, USA

**Keywords:** Delta large-channel endoscopy, lumbar decompression, lumbar spinal stenosis, minimally invasive spine surgery, endoscopic spine surgery, lumbar degenerative disease

## Abstract

**Highlights:**

**What are the main findings?**

- Current data suggest that Delta large-channel endoscopy may demonstrate comparable clinical efficacy and safety to microscopic, endoscopic, and open lumbar decompression techniques across pooled evidence.
- Some studies suggest select perioperative advantages relative to comparator approaches, including shorter hospital stay with comparable mid-term pain reduction, despite similar operative time and complication rates.

**What are the implications of the main findings?**

- Delta large-channel endoscopy may broaden the scope of minimally invasive spine surgery by facilitating adequate decompression of spinal stenosis through an expanded uniportal approach.
- The current evidence base is of low certainty and should be interpreted as hypothesis-generating. Multicenter studies with greater geographic diversity and longer-term follow-up are warranted to define clearer indications and benefits for patient outcomes.

**Abstract:**

**Background:** Delta large-channel endoscopic decompression is an emerging, minimally invasive approach for lumbar degenerative disease, but its comparative effectiveness and perioperative performance have not been comprehensively synthesized. We performed a systematic review and meta-analysis to evaluate clinical, functional, and safety outcomes of Delta large-channel endoscopy relative to established decompression techniques. **Methods:** PubMed, Embase, and Scopus were searched from database inception through July 2025 (PROSPERO #CRD420251107750). Peer-reviewed English-language studies reporting extractable outcomes after Delta large-channel endoscopic surgery in adults were included. Random-effects meta-analyses were used for pooled comparisons. **Results:** Nine studies met the inclusion criteria, comprising 737 patients overall, including 379 treated with Delta large-channel endoscopy and 358 treated with comparator procedures. Reported outcome assessment generally ranged from 1 week to 12 months postoperatively, or variable latest follow-up timepoints, and mean follow-up was inconsistently reported. All studies originated in China and included five retrospective cohorts, two randomized controlled trials, one prospective cohort, and one case series. The pooled mean operative time for Delta procedures was 88.28 min (95% CI, 79.24–97.31), pooled mean intraoperative blood loss was 24.58 mL (95% CI, 11.14–38.02), and pooled mean hospital stay was 4.59 days (95% CI, 2.87–6.31). Compared with microscopic, endoscopic, and open techniques, Delta large-channel endoscopy showed no statistically significant differences in operative time (MD = 6.95 min, 95% CI: −11.51–25.40 min; *p* = 0.4037), intraoperative blood loss (MD, −40.62 mL; 95% CI, −83.68–2.44; *p* = 0.0598), 3-month ODI change from baseline (MD, 1.26; 95% CI, −2.17–4.69; *p* = 0.3661), or complication rates (OR, 0.67; 95% CI, 0.40–1.12; *p* = 0.1094). Delta procedures were associated with shorter hospital stay (MD, −1.69 days; 95% CI, −2.83 to −0.56; *p* = 0.0122) and marginally greater improvement in six-month VAS low back pain change from baseline (MD, 0.28; 95% CI, 0.26–0.29; *p* = 0.0002), though clinically insignificant. The pooled complication rate for Delta procedures was 6.0% (95% CI, 4–10%) and reported rates of excellent or good MacNab outcomes ranged from 80 to 93% across Delta cohorts. **Conclusions:** Delta large-channel endoscopy may provide clinical and functional outcomes comparable to established decompression techniques, with similar safety and potential perioperative benefits, including shorter hospitalization. However, these findings are based on limited, low-certainty evidence with high heterogeneity and should be interpreted as exploratory rather than definitive. Additional multicenter studies with longer follow-up, broader geographic representation, and higher methodological quality are necessary before definitive conclusions regarding comparative effectiveness can be established.

## 1. Introduction

Lumbar degenerative spine diseases represent a major and growing global burden, affecting an estimated 250 million individuals worldwide [1] and constituting a leading cause of disability and functional impairment [2]. Conditions such as lumbar spinal stenosis (LSS) and lumbar disc herniation (LDH) commonly result in chronic pain and neurogenic claudication due to compression and irritation of neural elements. While conservative management is typically a first-line approach, a substantial proportion of patients ultimately require surgical intervention to achieve durable symptom relief [3]. As population aging accelerates globally, the demand for lumbar decompression procedures is expected to rise.

In parallel, the evolution of minimally invasive spine surgery (MISS) has reshaped the surgical management of lumbar degenerative disease [4,5]. Endoscopic spine surgery has demonstrated advantages in minimizing soft tissue disruption, reducing intraoperative blood loss, shortening hospital stay, and facilitating earlier mobilization compared with conventional open approaches [6]. Beyond limiting surgical exposure, MISS techniques also impact the perioperative neurobiological environment, such as reducing paraspinal muscle injury, attenuating inflammatory signaling, and modulating postoperative nociceptive pathways [7,8]. These effects are directly relevant to early pain trajectories, functional recovery, and overall neurologic outcomes.

Percutaneous endoscopic lumbar discectomy (PELD) represents a foundational advancement within MISS, enabling effective treatment of LDH while preserving surrounding structures and limiting operative morbidity [9]. However, early-generation percutaneous endoscopic systems were constrained by limited visualization and small working channels, restricting their applicability in cases requiring more extensive bony decompression, such as central canal or bilateral recess stenosis. As a result, these systems have historically been best suited for soft-disc pathology rather than complex degenerative stenosis or bony abnormalities.

**Figure 1:**
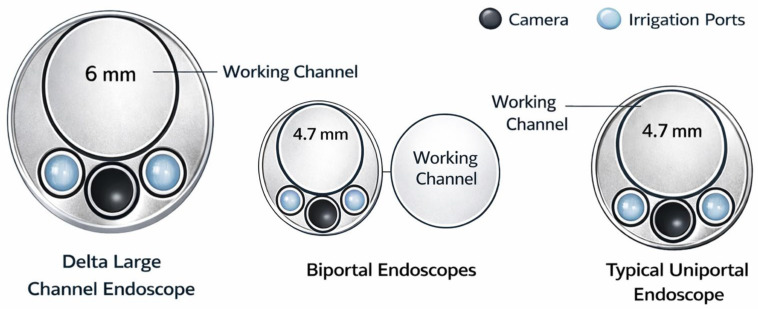
Cross-sectional view of Delta endoscope versus common comparator endoscopes.

Delta large-channel endoscopic spine surgery is a more recent endoscopic innovation designed to address these limitations. Developed over the last decade, the Delta system employs a uniportal endoscopic approach with an expanded working channel, approximately 6 mm in diameter, allowing for the use of larger-caliber instruments and facilitating more robust decompression maneuvers [10]. Unlike biportal endoscopic techniques, which separate visualization and instrumentation into two working portals, the Delta approach integrates both functions through a single channel [11]. This configuration enables central and bilateral decompression through a minimally invasive corridor, thereby extending the applicability of endoscopic surgery to pathologies traditionally managed with microscopic or open techniques, such as central canal and lateral recess stenosis [12,13]. A detailed cross-sectional comparison of endoscopic platforms—including the relative working channel diameters and spatial configuration of the camera and irrigation ports—is illustrated in Figure 1, highlighting the expanded working corridor afforded by the Delta large-channel endoscope compared with conventional uniportal and biportal systems.

Although early clinical studies have reported positive outcomes with Delta large-channel endoscopic spine surgery, the existing literature remains fragmented, and no prior systematic review has comprehensively synthesized the technique’s performance relative to established gold-standard approaches. The aim of this study is to systematically evaluate and quantitatively synthesize the available evidence on Delta large-channel endoscopy, with a focus on clinical efficacy, perioperative outcomes, and safety. By consolidating early clinical data, this review seeks to clarify the role of Delta large-channel endoscopy within contemporary MISS and its potential implications for patient recovery.

## 2. Methods

### 2.1. Systematic Literature Review

A systematic review and meta-analysis were conducted in accordance with the Preferred Reporting Items for Systematic Review and Meta-Analysis (PRISMA) guidelines. The study protocol was prospectively registered in PROSPERO (#CRD420251107750). A comprehensive literature search was performed in PubMed, Embase, and Scopus from database inception through July 2025. Search strategies incorporated controlled vocabulary and keyword combinations related to Delta large-channel endoscopy, endoscopic spine surgery, and lumbar degenerative disease. The complete search strategies for each database are provided in Supplementary Table S1.

Following the removal of duplicate records, all references were imported into Rayyan (Rayyan Systems Inc., Cambridge, MA, USA) for blinded screening.

### 2.2. Study Selection

Three reviewers (R.J., N.S., and M.M.) independently screened titles and abstracts for eligibility, followed by full-text review of potentially relevant studies. Studies were included if they were peer-reviewed, full-text articles published in English that enrolled adult patients undergoing spinal surgery using a Delta large-channel endoscopic approach and reported extractable clinical or perioperative outcomes.

Studies were excluded if they were cadaveric or biomechanical investigations, conference abstracts, review articles, or lacked relevant outcome data. Reference lists of included studies were manually screened to identify additional eligible publications. Any discrepancies during study selection were resolved through consensus discussion among the reviewers.

### 2.3. Data Extraction and Evidence Assessment

Data were extracted using a standardized data collection form and included patient demographics, surgical indications, operative time, intraoperative blood loss, incision length, length of hospital stay, pain and functional outcomes (Visual Analog Scale [VAS], Oswestry Disability Index [ODI], and MacNab scores reported as change from baseline where available), complication rates, and any reported cost-related or learning-curve variables.

Risk of bias was assessed using the RoB 2 tool for randomized controlled trials and the ROBINS-I tool for non-randomized comparative studies. Each study was independently assessed by three reviewers (R.J., M.M., and N.S.) using standardized signaling questions, with discrepancies resolved by consensus [14,15]. For the single case series included in the analyses, methodological quality was evaluated using the National Institutes of Health (NIH) Quality Assessment Tool for Case Series Studies [16].

Certainty of evidence for each primary outcome was also evaluated by two reviewers (R.J. and N.S.) using the GRADE framework [17]. Outcomes were assessed across the domains of risk of bias, inconsistency, indirectness, imprecision, and publication bias. Certainty was categorized as high, moderate, low, or very low according to the GRADE criteria. Assessments were performed at the outcome level across all included studies rather than at the individual study level.

### 2.4. Statistical Analysis

Meta-analyses were performed using R statistical software (version 4.4.3). Random-effects models were applied to all pooled analyses to account for anticipated clinical and methodological heterogeneity. Pooled estimates were reported for all variables, when possible, to summarize the overall direction and range of effects for preliminary data on Delta large-channel endoscopy; however, in cases of high heterogeneity, these estimates should be interpreted as exploratory and hypothesis-generating rather than definitive. Effect estimates were calculated as mean difference (MD) for continuous outcomes and odds ratios (ORs) for dichotomous outcomes, each reported with corresponding 95% confidence intervals (CIs). For VAS and ODI metrics, a positive MD indicated a greater degree of improvement in the Delta group. Statistical heterogeneity was quantified using the I^2^ statistic.

Prespecified subgroup analyses were conducted according to comparator procedure type (microscopic- including tubular, endoscopic, or open surgery). Statistical significance was defined as a two-sided *p*-value < 0.05. When necessary, sample means and standard deviations were estimated from median and interquartile ranges (IQR) using the method described by Wan et al. [18]. This estimation was applied to one included study, which reported variables of interest in terms of medians and IQR [19].

## 3. Results

### 3.1. Study Selection and Characteristics

**Figure 2:**
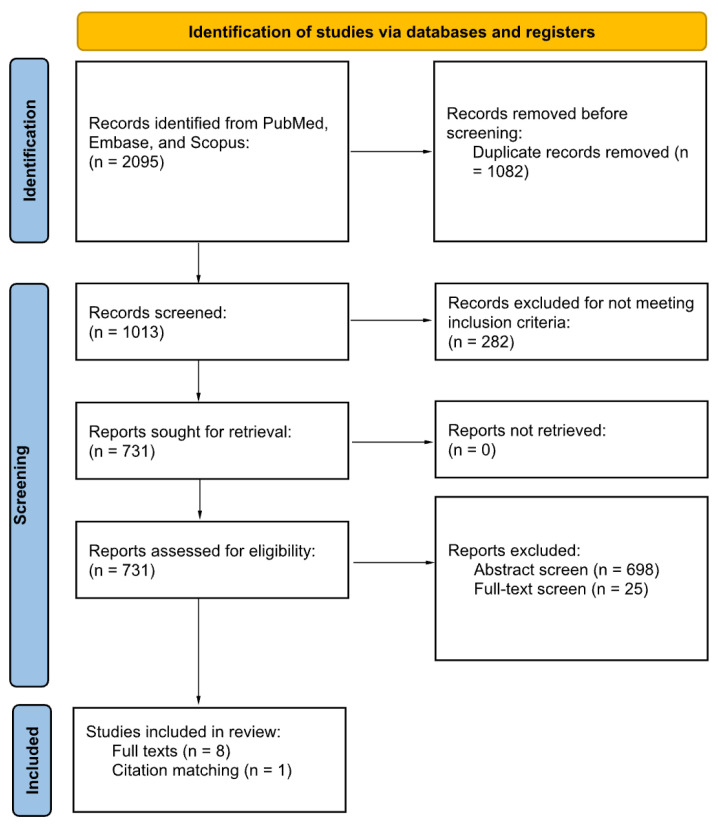
PRISMA diagram.

The search strategy initially yielded 2095 studies. After removal of duplicates (*n* = 1082) and studies that did not meet the inclusion criteria (*n* = 282) on the basis of title and source screening, 731 total studies were assessed for eligibility. Of these studies, 698 were removed based on abstract screening, and 25 were excluded upon full-text review. Eight studies were ultimately included following full-text review, and one additional study was retrieved through citation matching outside of the screening process (Figure 2) [10,12,19,20,21,22,23,24,25]. Given the limited published literature on Delta large-channel endoscopic spine surgery, all studies currently published on Delta endoscopic spine surgery were included; exclusions were only made if studies were not in English or not about the specified topic. This was solely a product of the sparse volume of literature available on the topic.

All included studies were published from 2020 onward and originated in China. Five studies (55.6%) were retrospective cohort studies, two studies (22.2%) were randomized controlled trials (RCTs), one study (11.1%) was a prospective cohort study, and one study (11.1%) was a case series. All studies except one (the case series) included a comparator procedure group: three microscopic [19,23,24], three endoscopic [10,12,21], and two open procedures [22,25].

The dominant surgical indication across the literature was lumbar spinal stenosis (LSS), reported in eight studies (88.9%), including those evaluating mixed degenerative disease. One study examined massive lumbar disc herniation (LDH) as the sole indication (11.1%), and one study included both LSS and LDH within the same cohort (11.1%).

### 3.2. Patient Demographics

Across included studies, 379 patients underwent Delta large-channel endoscopy surgery, while 359 patients underwent comparator procedures [10,12,19,21,22,23,24,25]; however, 737 patients were included in presented analyses due to the exclusion of one patient in Wei and colleagues’ work [10]. Mean age and sex distribution were comparable between the Delta large-channel endoscopy and comparator groups across included studies [10,12,19,20,21,23,24,25]. Reported follow-up (f/u) durations largely ranged from 1 week to approximately 26 months, with most studies reporting outcomes at 3-, 6-, and 12-month time points. Study characteristics are summarized in detail in Table 1 and Table 2. Of note, not all patients were captured in the meta-analyses, as one study in the literature review was a Delta-only case series without a comparator [20].

### 3.3. Risk of Bias and Certainty of Evidence Assessments

Risk of bias varied according to study design (Table 3a,b). Among randomized controlled trials, one study demonstrated some concerns using the RoB 2 tool, while the second was rated as being at “high risk of bias” due primarily to outcome measurement [10,22]. All non-randomized comparative studies were rated as being at “moderate risk” or “serious risk” of bias using ROBINS-I V2, largely due to confounding and outcome measurement domains (Table 3b). The single included case series was rated “good quality” using the NIH Quality Assessment Tool [20]. The GRADE assessment further demonstrated that the overall certainty of evidence ranged from low to very low across all primary outcomes (Table 4). Certainty was primarily limited by the predominance of non-randomized study designs and notable heterogeneity in several pooled analyses. Overall, the quality of evidence permits pooled analysis with explicit acknowledgment of study-level limitations that more strongly support hypothesis generation than conclusive evidence.

### 3.4. Cost Comparison

Two studies included reported hospitalization costs associated with Delta large-channel endoscopy. Wei et al. found significantly lower mean total cost in the Delta group (¥17,635.61 ± ¥3766.77 ≈ $2406.26 ± $513.59) compared with unilateral biportal endoscopy (¥31,091.69 ± ¥4222.64 ≈ $4244.01 ± $576.64; *p* < 0.001) [10]. Similarly, Zhu et al. reported lower hospitalization costs for Delta endoscopy (¥26,787 ≈ $3750 USD) compared with open fenestration discectomy (¥29,494 ≈ $4129 USD) [25]. All currency conversions were taken from the time of data extraction.

### 3.5. Perioperative Outcomes

A thorough overview of the included studies’ perioperative outcomes is included in Table 5.

#### 3.5.1. Operative Time

**Figure 3:**
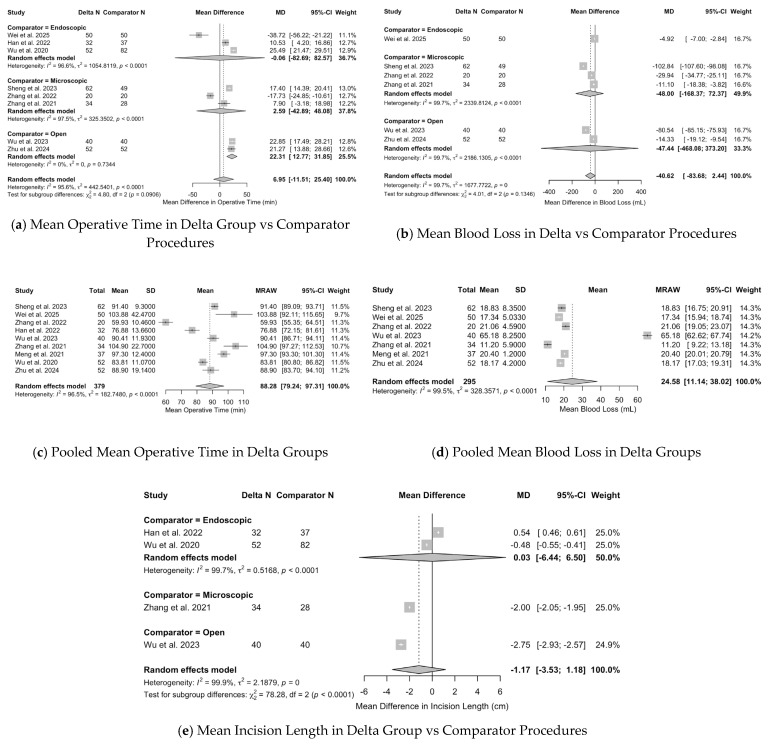
Forest plots of operative outcomes: (**a**) operative time, (**b**) intraoperative blood loss, and (**e**) incision length comparing Delta large-channel endoscopy with comparator procedures; (**c**) pooled mean operative time and (**d**) pooled mean intraoperative blood loss in Delta cohorts.

All included studies reported mean operative time [10,19,20,21,22,23,24,25]. The pooled mean operative time for Delta large-channel endoscopy across studies was 88.28 min (95% CI: 79.24–97.31 min; I^2^ = 96.5%) (Figure 3c). Pooled analysis demonstrated no significant difference in operative time between Delta and comparator procedures (MD = 6.95 min, 95% CI: −11.51–25.40 min; *p* = 0.4037; I^2^ = 95.6%), including across microscopic and endoscopic technique subgroups (Figure 3a). Pooled analysis showed a significantly higher operative time for Delta procedures relative to open comparator procedures (MD = 22.31 min, 95% CI: 12.77–31.85 min; *p* = 0.0214; I^2^ = 0%) (Figure 3a). Given the substantial heterogeneity, pooled estimates for operative time derived from random-effects models should be interpreted with caution. 

#### 3.5.2. Intraoperative Blood Loss

The pooled mean intraoperative blood loss for the Delta group across the seven studies that reported data was 24.58 mL (95% CI: 11.14–38.02 mL; I^2^ = 99.5%) [10,19,20,22,23,24,25] (Figure 3d). Pooled analysis across six studies with a comparator procedure and reported intraoperative blood loss showed no significant difference in intraoperative blood loss between Delta and comparator procedures (MD: −40.62 mL; 95% CI: −83.68–2.44 mL; *p* = 0.0598; I^2^ = 99.7%) (Figure 3b). The single study included in this analysis investigating an endoscopic comparator procedure showed a significant difference in intraoperative blood loss (MD: −4.92 mL; 95% CI: −7.00 to −2.84 mL, *p* < 0.0001) [10]. No significant difference in intraoperative blood loss was found between Delta and the microscopic comparator subgroup (MD: −48.00 mL; 95% CI: −168.37 to 72.37 mL; *p* = 0.2283; I^2^ = 99.7%) and no significant difference in intraoperative blood loss was found between Delta and the open comparator subgroup (MD: −47.44 mL; 95% CI: −468.08 to 373.20 mL; *p* = 0.3789; I^2^ = 99.7%) (Figure 3b). As with the operative time analyses, substantial between-study heterogeneity for intraoperative blood loss indicates that pooled estimates should be interpreted cautiously. Nonetheless, minimal blood loss is a consistent finding across all included studies; the combination of continuous irrigation and limited dissection likely contributes to this pattern, rendering Delta comparable to existing MISS approaches.

#### 3.5.3. Incision Length

Four studies reported incision length [12,21,22,24]. Pooled analysis demonstrated no significant difference in incision length between Delta and comparator procedures (MD: −1.17 cm; 95% CI: −3.53–1.18 cm; *p* = 0.2116) (Figure 3e). The single study included in this analysis investigating a microscopic comparator procedure showed a significant difference in incision length (MD: −2.00 cm; 95% CI: −2.05 to −1.95 cm; *p* < 0.0001), as did the single study included in this analysis investigating an open comparator procedure (MD: −2.75 cm; 95% CI: −2.93 to −2.57 cm; *p* < 0.0001). No significant difference was found between Delta and the endoscopic comparator subgroup (MD: 0.03 cm; 95% CI: −6.44 to 6.50 cm; *p* = 0.9683; I^2^ = 99.7%) (Figure 3e). Significant between-study heterogeneity was observed (I^2^ = 99.9%, *p* < 0.0001).

#### 3.5.4. Length of Hospital Stay

**Figure 4:**
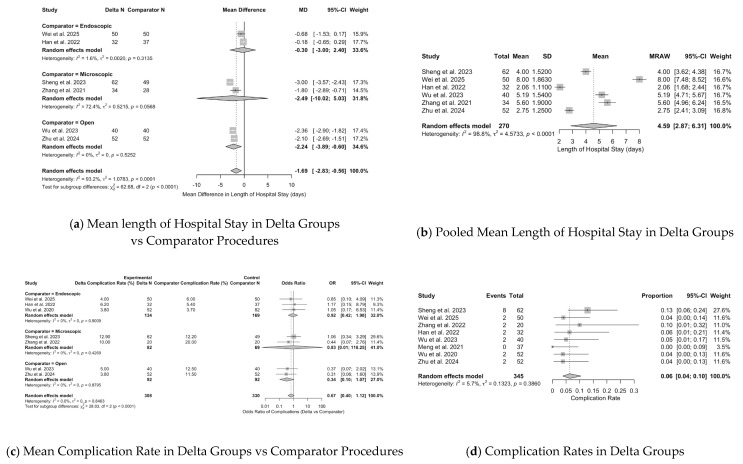
Forest plots of postoperative outcomes: (**a**) length of hospital stay and (**c**) complication rates comparing Delta large-channel endoscopy with comparator procedures; (**b**) pooled mean length of hospital stay and (**d**) pooled complication rate in Delta cohorts.

Six studies reported patients’ length of hospital stay [10,12,19,22,24,25]. The pooled mean length of stay for Delta large-channel endoscopy was 4.59 days (95% CI: 2.87–6.31 days; I^2^ = 98.8%) (Figure 4b). Comparative pooled analysis demonstrated a significantly shorter hospital stay for Delta procedures relative to comparator techniques (MD: −1.69 days; 95% CI: −2.83 to −0.56 days; *p* = 0.0122; I^2^ = 93.2%). Subgroup analyses showed significantly shorter hospital stays when compared with open comparator procedures (MD: −2.24 days; 95% CI: −3.89 to −0.60 days; *p =* 0.0367; I^2^ = 0%), with no significant difference observed relative to microscopic and endoscopic comparators (Figure 4a). Of note, discharge practices likely varied across institutions and healthcare systems, and given the geographic homogeneity of the included studies, these findings may not be widely applicable.

### 3.6. Clinical Outcomes

Postoperative outcomes are summarized according to short-term (≤1 week), mid-term (3–6 months), and longer-term (≥12 months) follow-up intervals.

#### 3.6.1. Pain Outcomes (VAS)

**Figure 5:**
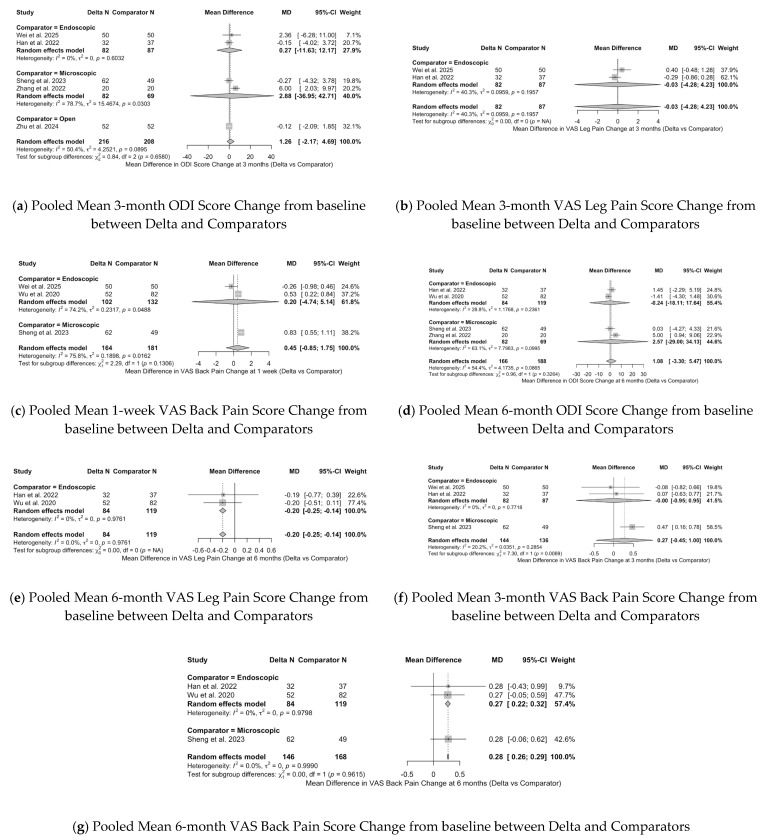
Forest plots of pooled mean differences in patient-reported outcomes: (**a**) 3-month ODI score change, (**b**) 3-month VAS leg pain score change, (**c**) 1-week VAS back pain score change, (**d**) 6-month ODI score change, (**e**) 6-month VAS leg pain score change, (**f**) 3-month VAS back pain score change, and (**g**) 6-month VAS back pain score change between Delta large-channel endoscopy and comparator procedures.

VAS back (VAS-LBP) and leg (VAS-LP) pain scores improved significantly from baseline in both Delta and comparator groups. However, no consistent between-group differences were reported at early postoperative time points, and most benefits were apparent during later observation periods (Figure 5b,c,e–g, Table 6).

VAS-LP analyses included three studies overall; 3-month VAS-LP was reported by Wei et al. and Han et al. [10,12], while 6-month VAS-LP was reported by Han et al. and Wu et al. [12,21]. No significant difference was found between Delta and endoscopic comparator groups for the three-month VAS-LP score change from baseline (MD: −0.03; 95% CI: −4.28–4.23; *p* = 0.9463; I^2^ = 40.3%). Pooled analysis revealed a significant difference between Delta and comparator groups for the six-month VAS-LP score change from baseline, indicating a greater degree of reduction in leg pain at six months in the endoscopic comparator group (MD: −0.20; 95% CI: −0.25 to −0.14; *p* = 0.0135; I^2^ = 0.0%). However, given the small MD, this difference is likely not clinically significant.

Four studies in total reported VAS-LBP at one week, three months, or six months postoperatively [10,12,19,21]. No significant difference was found between the Delta and comparator groups for the one-week VAS-LBP score change from baseline (MD: 0.45; 95% CI: −0.85–1.75; *p* = 0.2737; I^2^ = 75.8%). No significant difference was found between the Delta and comparator groups for the three-month VAS-LBP score change from baseline (MD: 0.27; 95% CI: −0.45–1.00; *p* = 0.2440; I^2^ = 20.2%). Pooled analysis revealed a significant difference between the Delta and comparator groups for the six-month VAS-LBP score change from baseline, indicating a greater degree of reduction in back pain at six months in the Delta group (MD: 0.28; 95% CI: 0.26–0.29; *p* = 0.0002; I^2^ = 0.0%). However, given the small MD, this difference between Delta and comparator groups is likely not clinically significant.

Across both back and leg pain scales, Delta cases demonstrated sustained symptom improvement. Several authors specifically attributed early pain reduction to smaller skin incisions and decreased muscle stripping [10,21,24]. Across mixed pathologies, Delta patients achieved similar or superior short-term pain relief compared with both endoscopic and microscopic techniques [10,12,19].

#### 3.6.2. Functional Outcomes (ODI)

ODI scores improved significantly from baseline in all groups. However, pooled analyses demonstrated no significant differences between Delta and comparator procedures for the three-month [10,12,19,23,25] (MD: 1.26; 95% CI: −2.17–4.69; *p* = 0.3661; I^2^ = 50.4%) [10,12,19,23,25] or six-month [12,19,21,23] postoperative ODI score change from baseline (MD: 1.08; 95% CI: −3.30–5.47; *p* = 0.4895; I^2^ = 54.4%) (Figure 5a,d; Table 6). In head-to-head study comparisons, Delta demonstrated comparable functional outcome improvement relative to microscopic and biportal techniques at 3–12 months. Studies with mixed stenosis and herniation populations also showed comparable functional recovery even in more complex decompressions, suggesting potential applicability across mixed degenerative pathologies.

#### 3.6.3. MacNab Criteria

Five studies also reported outcomes using the MacNab criteria for recovery of lumbar function (Table 6) [12,19,20,21,22].

Rates of excellent or good outcomes ranged from 80 to 93% in Delta cohorts and were comparable to comparator procedures at final follow-up.

### 3.7. Safety and Complications

Seven studies reported postoperative complications (Figure 4c,d) [10,12,19,21,22,23,25]. The pooled complication rate for Delta large-channel endoscopy was 6.0% (95% CI: 4–10%, I^2^ = 5.7%). Comparative analysis demonstrated no significant difference in complication rates between Delta and comparator procedures (OR = 0.67; 95% CI: 0.40–1.12; *p* = 0.1094; I^2^ = 0%) (Figure 4c).

Dural tears were the most frequently reported complication, occurring at similar frequencies in Delta and comparator groups (approximately 0–5%), but with no long-term sequelae [10,12,19,21,23]. No persistent cerebrospinal fluid leaks or procedure-related deaths were reported in the Delta cohorts, although transient nerve-related symptoms and isolated wound-related complications were reported [12,19,21,23,25]. Reoperation rates were low across studies, although long-term follow-up data are sparse.

## 4. Discussion

Delta large-channel endoscopic spine surgery represents a newer adaptation within MISS, combining single-portal endoscopic access with an expanded working channel that enables more extensive decompression than earlier full-endoscopic systems. This systematic review and meta-analysis is the first to synthesize available clinical evidence evaluating the efficacy, perioperative outcomes, and safety profile of Delta large-channel endoscopy relative to established endoscopic, microscopic, and open decompression techniques. Overall, the findings suggest that Delta large-channel endoscopy may achieve clinical and functional outcomes comparable to those of conventional approaches, while offering potential advantages in perioperative recovery, particularly shorter hospital stays. However, these findings should be interpreted cautiously and as exploratory, rather than definitive, given the current scope of the included studies.

Across included studies, Delta large-channel endoscopic surgery was most commonly executed via a unilateral interlaminar approach to achieve central or bilateral decompression [10,12,19,21,23,24,25]. The expanded working channel permits the use of standard decompression instruments, including drills and Kerrison rongeurs, facilitating adequate bony and ligamentous decompression through a minimally invasive corridor [24]. This technical capability may expand the range of targetable degenerative spine conditions, addressing the limitations of traditional percutaneous endoscopic systems that were historically reserved for soft-disc pathologies and microscopic or open approaches for central canal or bilateral recess stenosis [21,26].

Importantly, operative time did not differ significantly between Delta large-channel endoscopy and comparator procedures overall, although subgroup findings varied by comparator type. Delta procedures had comparable operative time relative to endoscopic and microscopic comparators but were associated with longer operative time than open comparator procedures. This finding should be interpreted cautiously given the limited evidence base, substantial statistical heterogeneity, variability in surgeon experience and clinical workflow, differences in case type, and the inherent learning curve associated with Delta large-channel endoscopy. Notably, the included open comparator procedures consisted of bilateral laminotomy [22] and fenestration discectomy [25], which are not necessarily equivalent to more extensive traditional open decompression procedures. With recent data suggesting that full-endoscopic cases may be associated with fewer transfusions and shorter hospital stays, Delta endoscopy may serve as a feasible alternative option in appropriately selected patients [27].

Even with streamlined surgical access, the aggregate evidence indicates that Delta large-channel endoscopy may achieve clinical outcomes comparable to other MISS techniques, with potential postoperative benefits [19,27]. For example, in a one-year cohort comparing Delta decompression to microscope-assisted laminectomy, both groups had significant pain relief and functional gains at 3-, 6-, and 12-month time points with no statistical difference in final outcomes [19]. Similarly, in a head-to-head randomized trial versus unilateral biportal endoscopy, Wei et al. found that the Delta approach yielded comparable clinical outcomes with benefits for perioperative efficiency, intraoperative blood loss, and improvement in lumbar function in the early postoperative period [10]. Although several studies reported significantly reduced immediate postoperative back pain and functional improvement with Delta, our pooled analyses only demonstrated clinically insignificant between-group differences at the six-month mark [19,21,23]. These findings suggest that the primary advantages of Delta may pertain to perioperative recovery rather than superior pain relief. It is further possible that the observed outcomes may reflect differences in tissue disruption and neural handling rather than decompression adequacy alone. Endoscopic approaches, including Delta large-channel endoscopy, minimize paraspinal muscle dissection and reduce iatrogenic soft-tissue injury, which may attenuate postoperative inflammatory responses and nociceptive sensitization [28]. These factors are likely contributors to early- or mid-term postoperative pain reductions reported in several included studies, albeit the pooled conclusions are not as robust. The exact mechanisms of these effects remain incompletely understood and speculative.

Length of hospital stay trended toward durations shorter in Delta cohorts, with the clearest pooled benefits observed relative to open comparator procedures; although the microscopic subgroup did not demonstrate a significant pooled difference, both studies individually favored shorter hospitalization after Delta endoscopy. Differences between Delta cohorts and comparators in length of stay overall were modestly significant; as such, studies suggest some reproducible perioperative benefit. However, evidence regarding fully ambulatory or same-day discharge pathways remains limited, and institutional discharge practices likely contributed to the noted statistical variability. Future studies are needed to define the role of Delta large-channel endoscopy within outpatient spine surgery paradigms.

Despite these favorable findings, several considerations warrant caution. Operative time and perioperative outcomes demonstrated substantial heterogeneity across studies, likely reflecting differences in surgeon experience, case selection, and institutional workflows [12,20,21,22,25]. As a result, pooled estimates should be viewed as hypothesis-generating summaries rather than precise estimates of comparative treatment effects, as they reflect an average effect across variable study populations and methodologies and may not represent a single underlying effect size. For instance, while some groups have noted no significant differences in mean operative time between Delta endoscopy and Quadrant tubular microsurgery, others have reported a significant improvement when compared to biportal endoscopy due to decreased instrument conflict [10,24]. Several authors have also commented on a learning curve associated with the Delta technique, which may influence operative efficiency and early outcomes during initial adoption [10,21]. As with other MISS approaches, outcomes are likely to be highly operator-dependent, and broader dissemination should be accompanied by structured training and competency frameworks. Similarly, cost-related conclusions are indeterminate; current data are limited to two independent studies and therefore insufficient to derive any conclusions related to cost-effectiveness.

Several limitations of this review must be acknowledged. Because Delta large-channel endoscopy remains an emerging technique with a relatively limited evidence base, this review reflects the currently available literature on the topic, all of which originated from China. Exclusion of non-English publications may have introduced language bias and may limit the generalizability of the present findings. Follow-up durations most often ranged from only 6 to 12 months; the GRADE assessment demonstrated low to very low certainty of evidence across outcomes (reflecting the predominance of non-randomized studies), and ROBINS-I and RoB 2 analyses noted consistent risk of bias within the available literature. High heterogeneity in perioperative outcome meta-analyses further limits the degree of interpretability of the effect sizes. As such, interpretation of pooled perioperative outcomes should be tempered, particularly for operative time, blood loss, and incision length. The variability in comparator techniques, surgeon learning curves (as a relatively new approach), pathology severity, and perioperative protocols likely contributed to the observed dispersion in effect estimates. Further, follow-up durations were relatively short, and long-term outcomes related to restenosis, spinal stability, and adjacent-segment degeneration remain insufficiently characterized.

Despite these limitations, the present studies suggest that Delta large-channel endoscopic decompression has meaningful technical and clinical potential. However, more rigorous studies are required to define its comparative efficacy, and current gaps in evidence are likely the result of limited study design rather than restricted access to the technology, which is already commercially available in the United States. Future research should focus on prospective, multicenter randomized studies comparing Delta large-channel endoscopy with both microscopic and open decompression techniques, particularly in diverse patient populations. Long-term follow-up is needed to assess the durability of decompression and potential late sequelae. In parallel, studies incorporating patient-reported outcome measures, health economic analyses, and learning curve assessments will be essential to define the optimal role of Delta large-channel endoscopy within modern spine care. These efforts will help delineate which outcomes are clinically meaningful.

## 5. Conclusions

Delta large-channel endoscopic spine surgery appears to be a promising minimally invasive option for lumbar decompression. Across the current literature synthesized in the presented study, Delta has achieved clinical and functional outcomes comparable to those of established microscopic, endoscopic, and open techniques, although the certainty of evidence is consistently low to very low. Across current published studies, the Delta approach demonstrated comparable pain relief, functional improvement, and safety profiles, with potential advantages in perioperative recovery metrics such as length of hospital stay. Notably, it may decompress central and foraminal stenosis while preserving stability, although long-term clinical outcomes remain uncertain. As surgical technology evolves, refined endoscopes with improved 3D visualization or specialized instrumentation may further streamline maneuverability through the uniportal corridor. This may enable more surgeons to adopt the method, particularly for ambulatory spine surgery, wherein operative efficiency and minimal soft-tissue disruption are most ideal.

While early clinical results are encouraging, the current evidence base is limited by restricted geographic concentration, heterogeneous study designs, and relatively short follow-up durations. Further prospective, multicenter investigations with longer-term follow-up, formal cost-effectiveness analyses, and standardized outcome reporting are needed to define the durability and optimal clinical indications for Delta large-channel endoscopy. As MISS continues to evolve, Delta large-channel endoscopy may play a role in the efficient and effective management of lumbar degenerative disease. Future studies are necessary to establish a stronger evidence base.

## Figures and Tables

**Table 1:** Included studies’ core characteristics.

Study ^+^	Year	Study Type	*n*	Indication	Comparator Procedure	Follow-Up Time, Mean ± SD (mo.)
Han et al. [12]	2022	Retrospective Cohort	Delta: 32 Comparator: 37	Lumbar central spinal stenosis	Endoscopic decompression	12 (SD NR)
Meng et al. [20]	2021	Case Series	Delta: 37	Massively prolapsed intervertebral disc herniation	None	Range: 7–13 (Mean and SD NR)
Sheng et al. [19]	2023	Prospective Cohort	Delta: 62 Comparator: 49	Lumbar spinal stenosis	Microscopic decompression	13.3 ± 4.3
Wei et al. [10]	2025	Randomized Controlled Trial	Delta: 50 Comparator: 50	Lumbar spinal stenosis	Endoscopic decompression	NR
Wu et al. [22]	2023	Randomized Controlled Trial	Delta: 40 Comparator: 40	Degenerative lumbar spinal stenosis	Open decompression	12 (SD NR)
Wu et al. [21]	2020	Retrospective Cohort	Delta: 52 Comparator: 82	Lumbar central spinal and lateral recess stenosis	Endoscopic decompression	Delta: 20.54 ± 5.49 Comparator: 21.22 ± 5.09
Zhang et al. [23]	2022	Retrospective Cohort	Delta: 20 Comparator: 20	Lumbar spinal stenosis	Microscopic decompression	6 (SD NR)
Zhang et al. [24]	2021	Retrospective Cohort	Delta: 34 Comparator: 28	Lumbar degenerative disease	Microscopic decompression (tubular)	NR
Zhu et al. [25]	2024	Retrospective Cohort	Delta: 52 Comparator: 52	Lumbar disc herniation	Open decompression	12 (SD NR)

Footnote: ^+^ All included studies were conducted in China; mo. = month; NR = not reported; SD = standard deviation.

**Table 2:** Included studies’ demographic characteristics.

	Delta Large Channel Endoscopy	Comparator Procedure
Weighted mean age ± pooled SD (years)	63.95 ± 10.75	63.22 ± 9.86
Female, *n* (%) ^+^	162 (47.78%)	156 (49.10%)
Total population, *n* **^++^**	379	359

Footnote: ^+^ Gender breakdown not reported by all included studies; presented out of available total. ^++^ Wei et al. randomized 51 comparator patients but excluded one from final analysis; outcome analyses reflect *n* = 50 for the comparator group when reported hereafter, though the present table includes this patient for demographic counts only (i.e., analyzed total *n* = 737); SD = standard deviation.

**Table 3:** (**a**) RoB 2 risk of bias assessment for included randomized controlled trials. Domains are: 1 (randomization), 2 (deviations), 3 (missing data), 4 (measurement), 5 (selection). (**b**) ROBINS-I V2 risk of bias assessment for included non-randomized, comparative studies. Domains are: 1 (confounding), 2 (selection), 3 (classification), 4 (missing data), 5 (measurement), and 6 (selection).

(**a**) **RoB 2 Assessment**							
**Author (Year)**	**D1**	**D2**	**D3**	**D4**	**D5**	**Overall Risk**	**Predicted Direction**
Wu et al. (2023) [22]	Some concerns	Some concerns	Low risk	High risk	Some concerns	Some concerns/high risk	Favors Delta
Wei et al. (2025) [10]	Low risk	Low risk	Low risk	Low risk	Some concerns	Some concerns	Favors Delta
(**b**) **ROBINS-I V2 Assessment**							
**Author (year)**	**D1**	**D2**	**D3**	**D4**	**D5**	**D6**	**Overall risk**
Han et al. (2022) [12]	Serious	Low	Moderate	Moderate	Moderate	Moderate	Moderate
Sheng et al. (2023) [19]	Serious	Low	Serious	Serious	Moderate	Moderate	Serious
Wu et al. (2020) [21]	Serious	Low	Moderate	Moderate	Moderate	Moderate	Moderate
Zhang et al. (2022) [23]	Serious	Low	Moderate	Moderate	Moderate	Moderate	Moderate
Zhang (2021) [24]	Serious	Low	Moderate	Moderate	Moderate	Moderate	Moderate
Zhu et al. (2024) [25]	Moderate	Low	Moderate	Moderate	Moderate	Moderate	Moderate

**Table 4:** Certainty of evidence and study outcomes summary table. GRADE evaluation entails assessment of key outcomes across the sum of evidence included in the review and analysis.

Outcome	Effect Estimate (95% CI)	Studies, *n*	Total Patients Included, *n*	Certainty
Operative Time	MD 6.95 (−11.51–25.40)	8	700	Very low
Blood Loss	MD −40.62 (−83.68–2.44)	6	497	Very low
Incision Length	MD −1.17 (−3.53–1.18)	4	345	Very low
Length of Stay	MD −1.69 (−2.83 to −0.56)	6	526	Low
ODI 3 mo.	MD 1.26 (−2.17–4.69)	5	424	Low
ODI 6 mo.	MD 1.08 (−3.30–5.47)	4	354	Low
VAS BP 1 wk.	MD 0.45 (−0.85–1.75)	3	345	Very low
VAS BP 3 mo.	MD 0.27 (−0.45–1.00)	3	280	Low
VAS BP 6 mo.	MD 0.28 (0.26–0.29)	3	314	Low
VAS LP 3 mo.	MD −0.03 (−4.29–4.23)	2	169	Very low
VAS LP 6 mo.	MD −0.20 (−0.25 to −0.14)	2	203	Low
Complications	OR 0.67 (0.40–1.12)	7	638	Low

Footnote: MD = Mean difference; BP = back pain; LP = leg pain. Table excludes Meng et al. case series for outcomes.

**Table 5:** Summary of perioperative outcomes in the included studies.

Study (Year)	Mean Operative Time ± SD (min)	Mean Intraoperative Blood Loss ± SD (mL)	Mean Length of Hospital Stay ± SD (Days)
Sheng et al. (2023) [19]	Delta: 91.4 ± 9.3 Comparator: 74.00 ± 6.87 *p* < 0.05	Delta: 18.83 ± 8.35 Comparator: 121.67 ± 15.28 *p* < 0.05	Delta: 4 ± 1.52 Comparator: 7 ± 1.53 *p* < 0.05
Wei et al. (2025) [10]	Delta: 103.88 ± 42.47 Comparator: 142.60 ± 46.71 *p* < 0.001	Delta: 17.34 ± 5.03 Comparator: 22.26 ± 5.55 *p* < 0.001	Delta: 8.00 ± 1.86 Comparator: 8.68 ± 2.45 *p* = 0.121
Zhang et al. (2022) [23]	Delta: 59.93 ± 10.46 Comparator: 77.66 ± 12.44 *p* < 0.001	Delta: 21.06 ± 4.59 Comparator: 51.00 ± 10.02 *p* < 0.001	NR
Han et al. (2022) [12]	Delta: 76.88 ± 13.66 Comparator: 66.35 ± 13.05 *p* < 0.005	NR	Delta: 2.06 ± 1.11 Comparator: 2.24 ± 0.83 *p* = 0.442
Wu et al. (2023) [22]	Delta: 90.41 ± 11.93 Comparator: 67.56 ± 12.51 *p* < 0.001	Delta: 65.18 ± 8.25 Comparator: 145.72 ± 12.36 *p* < 0.001	Delta: 5.19 ± 1.54 Comparator: 7.55 ± 0.83 *p* < 0.001
Zhang et al. (2021) [24]	Delta: 104.9 ± 22.7 Comparator: 97.0 ± 21.7 *p* = 0.165	Delta: 11.2 ± 5.9 Comparator: 22.3 ± 18.9 *p* = 0.005	Delta: 5.6 ± 1.9 Comparator: 7.4 ± 2.4 *p* = 0.002
Meng et al. (2021) [20]	Delta: 97.3 ± 12.4	Delta: 20.4 ± 1.2	NR
Wu et al. (2020) [21]	Delta: 83.81 ± 11.07 Comparator: 58.32 ± 12.30 *p* < 0.05	NR	NR
Zhu et al. (2024) [25]	Delta: 88.90 ± 19.14 Comparator: 67.63 ± 19.32 *p* < 0.0001	Delta: 18.17 ± 4.20 Comparator: 32.50 ± 17.13 *p* < 0.0001	Delta: 2.75 ± 1.25 Comparator: 4.85 ± 1.78 *p* < 0.0001

Footnote: NR = not reported; SD = standard deviation.

**Table 6:** Overview of included studies’ functional outcomes.

Study (Year)	VAS Low Back Pain (Preop. to Final f/u)	VAS Leg Pain (Preop. to Final f/u)	ODI (Preop. to Final f/u)	MacNab Criteria
Sheng et al. (2023) [19]	LBP (12 mo.): Delta: 6.42 ± 0.67 to 1.98 ± 1.55 (*p* < 0.05) Comparator: 6.19 ± 0.75 to 2.11 ± 1.62 (*p* < 0.05)	LP (12 mo.): Delta: 5.95 ± 0.87 to 2.07 ± 1.93 (*p* < 0.05) Comparator: 6.09 ± 0.84 to 1.82 ± 1.19 (*p* < 0.05)	12 mo.: Delta: 58.86 ± 5.64 to 27.90 ± 13.54 (*p* < 0.05) Comparator: 57.82 ± 6.16 to 29.29 ± 13.28 (*p* < 0.05)	Delta (12 mo.): 83.87% Comparator (12 mo.): 85.71% *(p* > 0.05)
Wei et al. (2025) [10] ^a^	LBP (3 mo.): Delta: 6.94 ± 2.02 to 1.46 ± 0.50 (*p* < 0.05) Comparator: 7.08 ± 2.14 to 1.52 ± 0.51 (*p* < 0.05)	LP (3 mo.): Delta: 6.68 ± 2.42 to 1.08 ± 0.27 (*p* < 0.05) Comparator: 6.38 ± 2.34 to 1.18 ± 0.39 (*p* < 0.05)	3 mo.: Delta: 65.56 ± 25.94 to 4.56 ± 10.33 Comparator: 65.44 ± 24.72 to 6.80 ± 13.32	NR
Zhang et al. (2022) [23] ^b^	Unspecified (6 mo.): Delta: 6.05 ± 1.19 to 1.25 ± 0.44 (*p* < 0.05) Comparator: 6.40 ± 1.47 to 1.30 ± 0.57 (*p* < 0.05)		6 mo.: Delta: 78.0 ± 7.0 to 21.0 ± 7.0 Comparator: 74.0 ± 7.0 to 22.0 ± 4.0	NR
Han et al. (2022) [12] ^c^	LBP (12 mo.): Delta: 5.25 ± 1.63 to 1.88 ± 0.98 (*p* < 0.05) Comparator: 5.03 ± 1.77 to 1.97 ± 1.09 (*p* < 0.05)	LP (12 mo.): Delta: 7.28 ± 1.02 to 1.56 ± 1.24 (*p* < 0.05) Comparator: 7.43 ± 1.21 to 1.49 ± 1.26 (*p* < 0.05)	12 mo.: Delta: 53.25 ± 7.70 to 18.19 ± 7.84 (*p* < 0.05) Comparator: 52.38 ± 8.22 to 19.24 ± 9.13 (*p* < 0.05)	Delta: 81.25% Comparator: 81.08% *(p* = 0.773)
Wu et al. (2023) [22]	VAS improved significantly in both groups postoperatively; lower in the Delta group at 1 week (*p* < 0.05), with no differences at later follow-up.		ODI improved significantly in both groups postoperatively; lower in the Delta group at 1 week (*p* < 0.05), with no differences at later follow-up.	Delta: 92.50% Comparator: 87.50% *(p* = 0.70)
Zhang et al. (2021) [24] ^d^	Unspecified (1 mo.): Delta: 7.1 ± 1.5 to 2.0 ± 0.7 Comparator: 6.6 ± 1.3 to 1.9 ± 0.8		1 mo.: Delta: 47.6 ± 10.7 to 8.4 ± 2.5 Comparator: 49.4 ± 12.7 to 10.8 ± 3.1	NR
Meng et al. (2021) [20]	LBP (6 mo.): Delta: 3.44 ± 0.85 to 0.55 ± 0.19	LP (6 mo.): Delta: 6.80 ± 0.73 to 0.25 ± 0.17	6 mo.: Delta: 60.2 ± 7.3 to 17.9 ± 3.4	Delta: 86.40%
Wu et al. (2020) [21] ^e^	LBP (variable last f/u): Delta: 5.13 ± 1.03 to 1.58 ± 0.70 Comparator: 4.93 ± 1.04 to 1.52 ± 0.76	LP (variable last f/u): Delta: 7.71 ± 0.91 to 1.62 ± 0.74 Comparator: 7.95 ± 0.99 to 1.71 ± 0.74	Variable last f/u: Delta: 74.62 ± 9.12 to 26.71 ± 6.45 Comparator: 76.90 ± 9.43 to 28.15 ± 6.59	Delta: 90.4% Comparator: 89.0%
Zhu et al. (2024) [25]	VAS scores were significantly lower in the Delta group at 24, 48, 72 h (*p* < 0.001, *p* = 0.01, *p* < 0.001)		12 mo.: Delta: 25.23 ± 5.90 to 2.04 ± 4.98 Comparator: 26.00 ± 5.73 to 3.17 ± 6.75	NR

Footnote: Follow-up times refer to the final reported assessment when available; early or nonstandard timepoints are explicitly labeled in individual cells. Reported *p*-values reflect source-reported within-group or between-group comparisons as specified for each study. Values are presented as mean ± standard deviation (SD). VAS = Visual Analog Scale; ODI = Oswestry Disability Index; LBP = low back pain; LP = leg pain; NR = not reported; mo. = month. ^a^ No between-group differences in VAS for low back pain and leg pain. Delta group demonstrated significantly lower ODI scores than the comparator group across all follow-up times. ^b^ No significant between-group differences in VAS at baseline or any follow-up time points. No significant between-group differences in ODI at most time points, except at 3 days postoperatively, where the observation group had lower ODI (0.33 ± 0.06 vs. 0.37 ± 0.05; *p* = 0.022). ^c^ No significant between-group differences in VAS or ODI throughout follow-up. ^d^ No significant between-group differences in VAS at baseline or 30 days; ODI was lower in the Delta group at 1 month. ^e^ Delta group showed lower VAS and ODI at 1 week (*p* < 0.05), with no significant differences at later follow-up time points.

## Data Availability

No new data were created. Data analyzed in this review are available from the cited studies and in presented materials.

## Supplementary Materials

The following supporting information can be downloaded at: https://www.mdpi.com/article/10.3390/brainsci16070731/s1, Table S1: Search terms used for literature search.
