# Supplementary material for: Clinical and Functional Outcomes of Delta Large-Channel Endoscopic Lumbar Decompression: A Systematic Review and Meta-Analysis
Source: Brain Sci. 2026 Jul 11;16(7):731. doi: 10.3390/brainsci16070731 (PMC13406147; doi:10.3390/brainsci16070731)
Supplement: Supplementary file 1 [file brainsci-16-00731-s001.zip › brainsci-4242845-supplementary.pdf]

Table S1. Search terms used for literature search.

Database	Search terms
**PubMed**	"Delta large channel" OR "large channel endoscopic" OR "endoscopic spine surgery" OR "delta endoscopic") AND (spine OR spinal OR lumbar OR thoracic OR cervical) AND (surgery OR discectomy OR decompression OR foraminotomy OR fusion)
**Embase**	('Delta large channel':ti,ab,kw OR 'large channel endoscopic':ti,ab,kw OR 'endoscopic spine surgery':ti,ab,kw OR 'delta endoscopic':ti,ab,kw)<br>AND<br>(spine:ti,ab,kw OR spinal:ti,ab,kw OR lumbar:ti,ab,kw OR thoracic:ti,ab,kw OR cervical:ti,ab,kw)<br>AND<br>(surgery:ti,ab,kw OR discectomy:ti,ab,kw OR decompression:ti,ab,kw OR foraminotomy:ti,ab,kw OR fusion:ti,ab,kw)
**Scopus**	(TITLE-ABS-KEY("Delta large channel" OR "large channel endoscopic" OR "endoscopic spine surgery" OR "delta endoscopic"))<br>AND<br>(TITLE-ABS-KEY(spine OR spinal OR lumbar OR thoracic OR cervical))<br>AND<br>(TITLE-ABS-KEY(surgery OR discectomy OR decompression OR foraminotomy OR fusion))
